# Case report: Surgical removal of an intradural and intramedullary brainstem foreign body in a young German Shepherd

**DOI:** 10.3389/fvets.2023.1123304

**Published:** 2023-03-16

**Authors:** Oscar Thamar-Torres, Andy Shores

**Affiliations:** ^1^Especialidades Centro Veterinario, Guatemala City, Guatemala; ^2^Department of Clinical Sciences, Neurosurgery/Neurology Section, Mississippi State University College of Veterinary Medicine, Mississippi State, MS, United States

**Keywords:** tetraparesis, brainstem foreign body, ventral craniectomy, 3D printing, intradural, intramedullary, brainstem

## Abstract

A young, female German Shepherd was presented for evaluation of a progressive, mildly ambulatory tetraparesis with severe neck pain. All segmental reflexes were intact, and the paresis was more severe on the right thoracic and pelvic limbs. Diagnostic imaging (radiographs and computed tomography) revealed 2 metallic linear foreign bodies lodged at the right side of the cervicomedullary junction. A modification of the previously described ventral craniectomy approach was performed and after removal of a portion of the basioccipital bone using a nitrogen powered drill, the foreign bodies were removed. Over a period of 3 months, the patient made a full recovery.

## Case description

A nine-month-old female German Shepherd was presented as an emergency for evaluation of a progressive, right asymmetrical, non-ambulatory tetraparesis and cervical pain. The dog had originally presented to its primary veterinarian at 7–1/2 months of age for anorexia and a yellow-tinged bilateral eye discharge. A diagnosis of Babesiosis was made based on peripheral blood smears (1) and treatment with doxycycline was initiated for a course of 35 days.

At the end of 35 days the patient was somewhat improved but had developed a dry cough and at times had difficulty swallowing. Thoracic radiographs were unremarkable and a diagnosis of laryngopharyngitis and possibly a mild esophagitis from the prolonged doxycycline therapy was made. Treatment consisted of five more days of oral doxycycline and carprofen.

The owners initially noted a slight improvement; however, 10 days later the dog would cry out in pain anytime the neck was moved, and she developed a progressive tetraparesis that was more pronounced on the right. This later development prompted the emergency clinic visit.

The neurologic examination revealed severe neck pain and a non-ambulatory tetraparesis with dysfunction of the right thoracic and pelvic limbs worse. Segmental reflexes were intact in all limbs and the lesion was localized to the upper cervical spine or lower brainstem. The diagnostic plan consisted of a CBC, cervical radiographs, a computed tomography (CT) scan of the head and cervical region and, possibly a cisternal CSF tap.

The CBC revealed an inflammatory leukogram (WBC 27,200; Neutrophils 22,774, and normal absolute lymphocytes and monocytes). The patient was sedated (dexmedetomidine 5 ug/ kg IV plus butorphanol 0.2 mg/kg IV) for lateral and ventrodorsal cervical radiographs to be followed by the CT scan.

**Figure 1:**
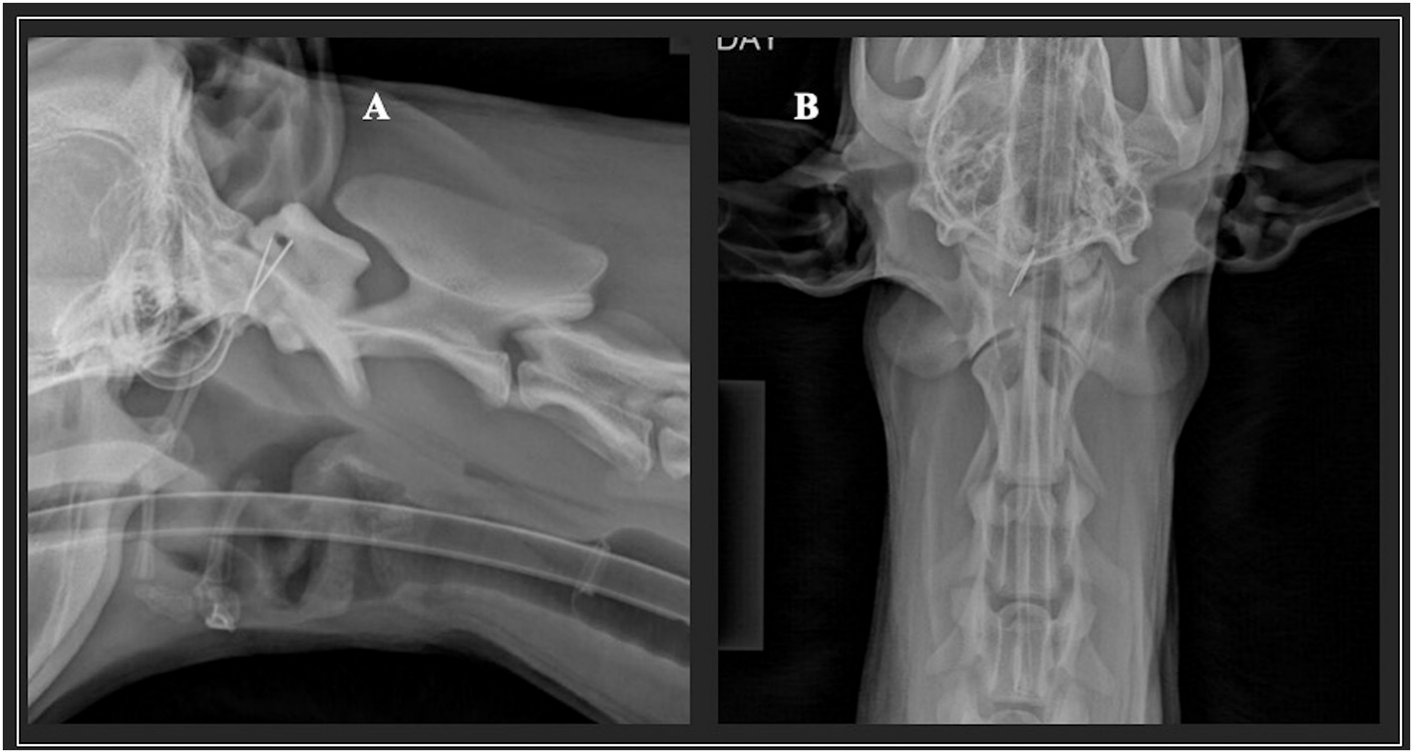
Lateral **(A)** and ventrodorsal **(B)** radiographs of a nine-month-old female German Shepherd was presented as an emergency for evaluation of a progressive, right asymmetrical, non-ambulatory tetraparesis and cervical pain. The metallic foreign bodies are very evident.

**Figure 2:**
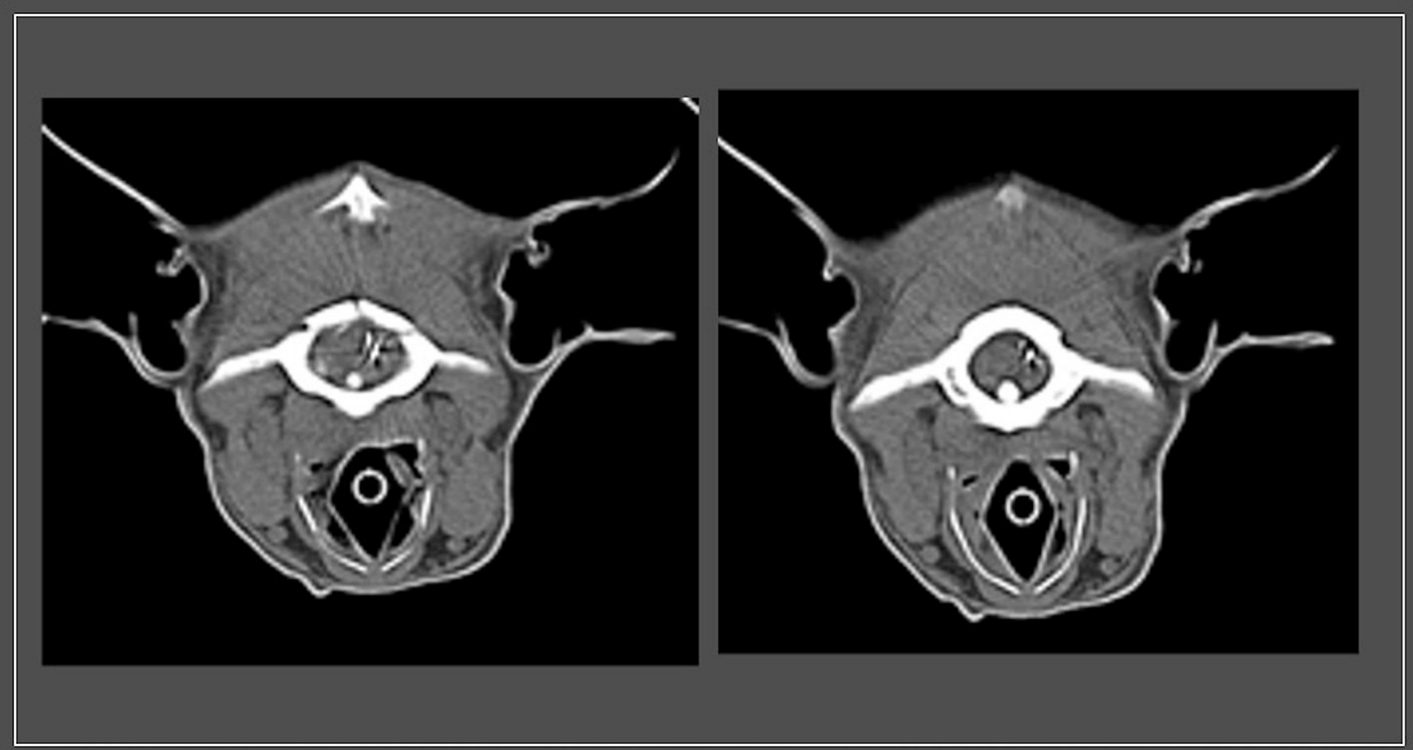
Transverse slices from computed tomography imaging at the region of the cervicomedullary junction showing metallic foreign bodies that are penetrating the dura at the level of the caudal brainstem (cervicomedullary junction).

Radiographs revealed two metallic foreign bodies lodged at the cervicomedullary junction (Figure 1). The CT was performed with the patient positioned in ventral recumbency. The CT delineated the foreign bodies were at least partially intradural and located on the right side of the cervicomedullary junction (Figure 2). Based on these findings, the progressive neurologic signs, and a concern for further migration of the foreign bodies, surgical removal was recommended. Based on the location of the foreign bodies, a ventral craniectomy was recommended for removal of the foreign bodies.

The advanced imaging provided the information that the location of the foreign bodies matched the neurologic localization. The presence of a migrating metallic foreign body in the brainstem does not have a therapeutic remedy other than surgical intervention as the concern was with additional migration and inflammatory response could result in death of the patient. Potential complications of the surgery included additional brainstem damage and swelling and damage to the basilar artery—both of which could have resulted in death of the patient.

**Figure 3:**
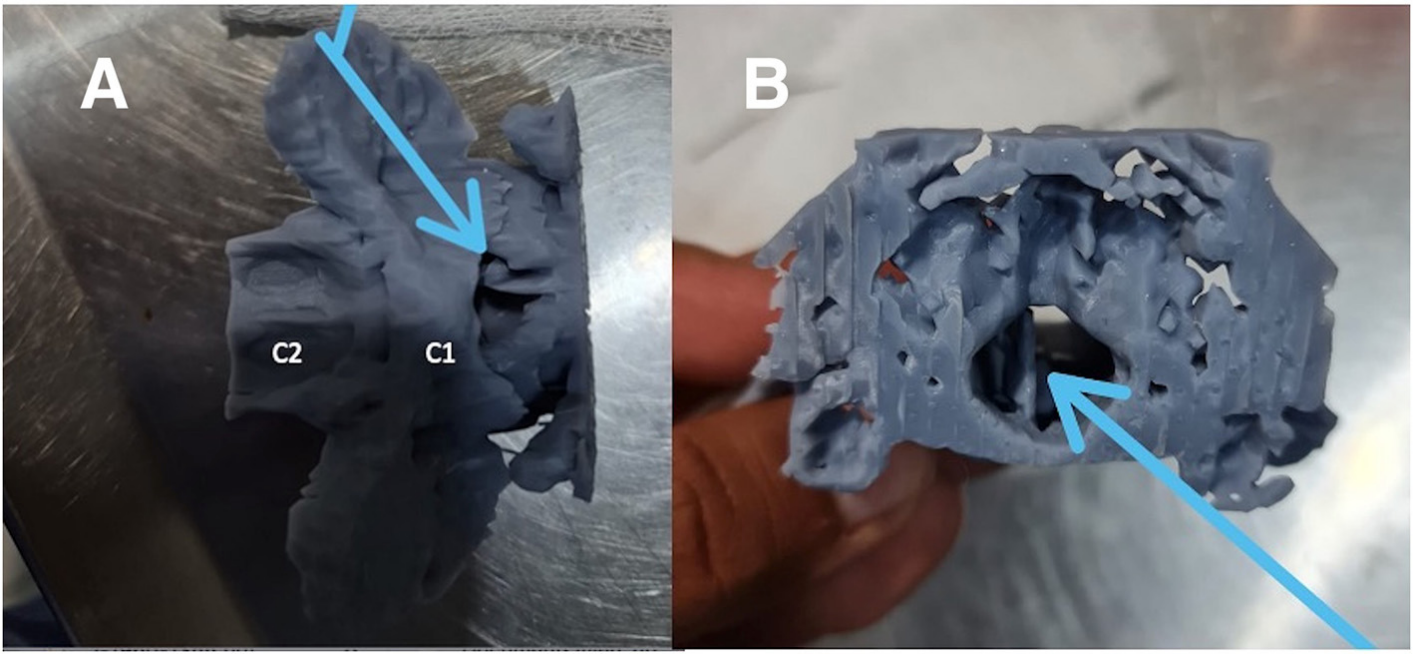
Dorsal **(A)** and cranial **(B)** view of the 3D resin print of the caudal portion of the occipital bone and the C1 and C2 vertebrae. The blue arrows point to the metallic foreign bodies.

In planning for the surgery, a 3D model of the cervicomedullary just was printed using a resin printer.^1^ An.STL file was made from the 3D reconstruction of the CT DICOM images and this file was used to make the print. The slice thicknesses of the CT scan were 2 mm with a 1 mm slice interval. These settings were more than adequate for a quality 3D reconstruction used for the 3D print file. This was valuable in planning the procedure and locating the exact position of the two metallic foreign bodies (Figure 3). The 3D print gave the surgeon an opportunity to view the exact location of the foreign bodies prior to the actual surgery.

The dog was premedicated with midazolam (0.3 mg/kg IV), ketamine (1.5 mg/kg IV, lidocaine 1.5 mg/kg IV) and anesthesia was induced with propofol (3 mg/kg IV). After tracheal intubation, the patient was maintained in a surgical plane of anesthesia on inhalation with 100% oxygen and 1.5−2.0 % isoflurane. SpO_2_, ETCO_2_, non-invasive blood pressure, and ECG were monitored constantly throughout the procedure.

The anesthetized patient was positioned in dorsal recumbency with the head and neck in maximal extension. An area from the middle of the mandible to the manubrium was clipped and prepped sterilely for surgery.

**Figure 4:**
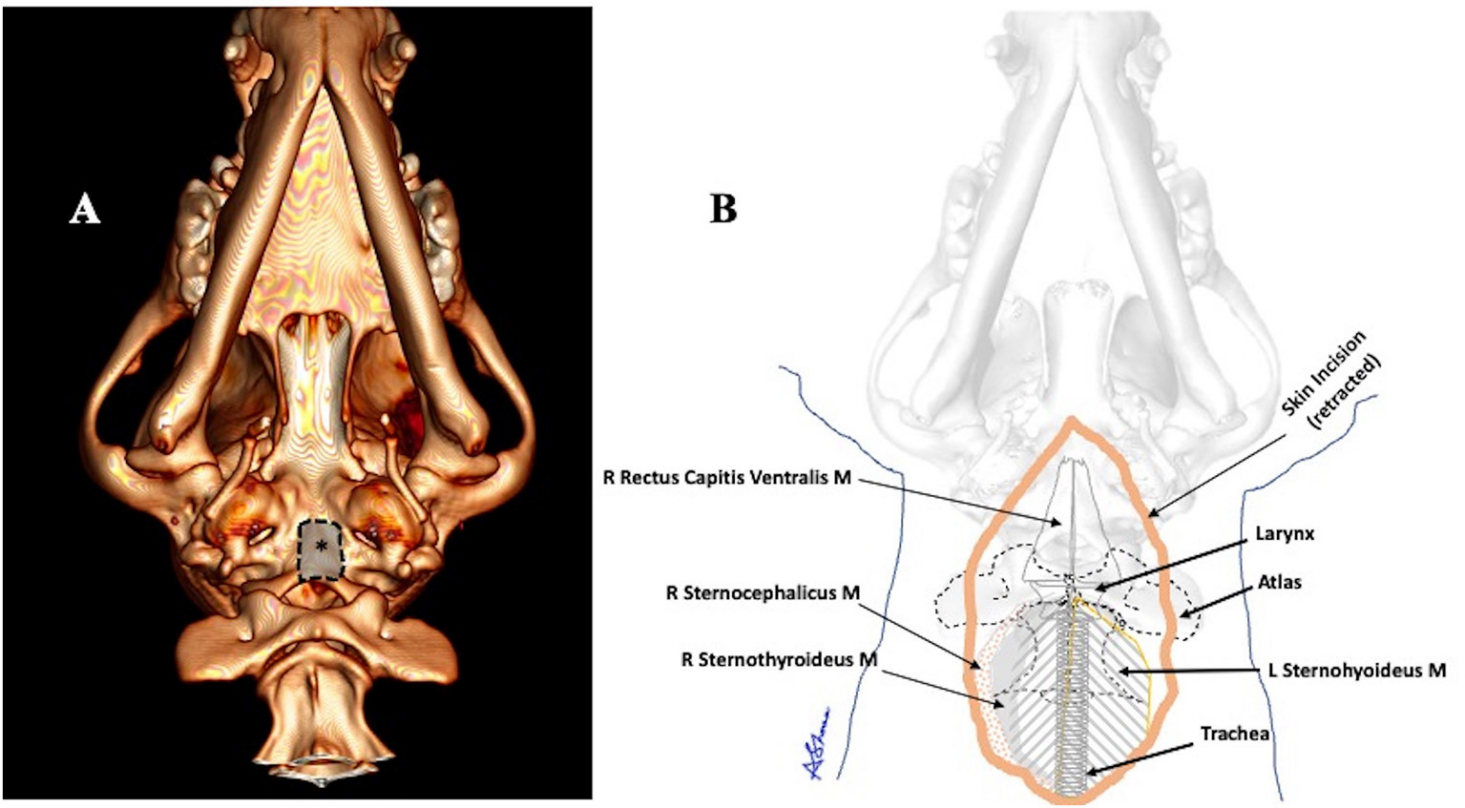
**(A)** A graphic representation of the area where the ventral craniectomy was performed (asterisk). **(B)** An illustration of showing many of the important anatomical structures encountered in this modified approach.

A ventral midline incision was made from the caudal one-third of the mandible to a point beyond the larynx. The ventral craniectomy was performed as previously described by Klopp et. al. (2) with slight modifications. After the skin incision, a paramedian approach was made and the right sternothyroideus and sternocephalicus muscles and right carotid sheath were separated by blunt dissection. Next, the right sternothyroideus muscle and right carotid sheath were retracted toward midline. This exposed the longus colli muscles overlying the ventral midline of the cervical vertebrae. After identifying the sharp ventral protuberance of the C1 vertebrae, midline muscle dissection/separation was made rostral to that point, exposing the ventral aspect of the atlanto-occipital joint. The rectus capitis ventralis and longus capitis muscles were identified and sharply dissected on the midline to expose the basioccipital bone. Gelpi retractors were used to maintain the exposure of the basioccipital bone, using care to not damage the internal carotid artery. An illustration of the pertinent anatomical structures associated with this approach is shown in Figure 4.

The basioccipital bone was drilled using a nitrogen powered drill^2^, drilling through the outer cortical and the medullary bone. Once the thin, white inner cortical layer was reached, drilling was stopped and Kerrison rongeurs were used to remove the thin shell of bone overlying the ventral medulla. The area opened with the ventral craniectomy is shown in Figure 4.

**Figure 5:**
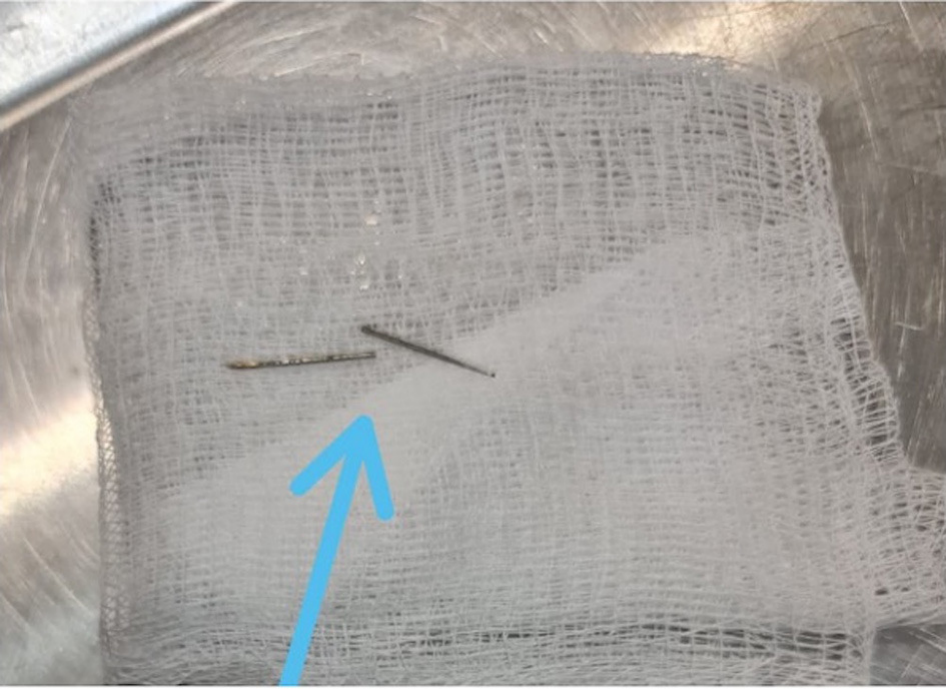
The blue arrow points to the 2 metallic objects retrieved in surgery that penetrated the dura and were partially lodged in the parenchyma of the brainstem. Each measured ~ 20 mm (0.75 inches) in length.

After exposing the ventral aspect of the medulla, a slightly blood-tinged fluid emerged from the site and two metallic objects were observed. The objects had penetrated the dura and were apparently lodged into the parenchyma of the medulla. These were carefully grasped and removed using small, curved hemostats. Examination of the metallic objects revealed they were two-halves of the shaft portion of a 40 mm (1.5 inch) hypodermic needle (Figure 5).

After copious flushing of the craniectomy site with warmed normal saline, the opening was covered with a sheet of gelatin sponge material.^3^ Muscles over the basioccipital bone, the longus colli muscles and the sternothyroideus and sternocephalicus muscles were approximated using a 3–0 synthetic resorbable suture. The subcutaneous tissue and skin were closed in a routine fashion. The patient recovered from anesthesia uneventfully.

The patient remained in the hospital for convalescence and physical therapy for nine days. Post-operative mediations included antibiotics (ceftriaxone 20 mg/kg IV, BID for 9 days) and analgesics (carprofen 2.2 mg/kg BID). Over a period of 2 weeks, the patient's neurologic status continued to improve and at 3 months post-operative, the neurologic examination was normal, and the owners reported no pain associated with the neck.

## Discussion

In a young dog presented with a history of progressive, asymmetrical tetraparesis and neck pain, the more typical rule outs are vertebral anomalies, infectious/inflammatory diseases (meningoencephalitis of unknown origin, infectious meningitis, diskospondylitis), and traumatic injuries such as a vertebral luxation. In addition, unusual and reported presentations have included aberrant parasite migration (*Dirofilaria immitis*) (3) and migrating foreign bodies from the oropharyngeal penetrating injuries. Most of the migrating foreign bodies are either of plant origin or pieces of wood (4). Metallic foreign bodies (sewing needles) that migrated into the central nervous system in dogs and cats are reported by several authors (5–7). Reports of surgical removal of these foreign bodies were mostly through an oral/pharyngeal approach. Removal of a metallic foreign body through a rostrotentorial craniectomy is reported and was after an unsuccessful attempt to remove it through the oral cavity (8).

The containment of an entire metallic foreign body within the calvarium removed by any form of a craniectomy has not been previously reported in the veterinary literature^.^ The ventral craniectomy is seldom indicated and the previously reported technique does require dissection around several vital structures. Our modification of the surgical procedure exposed the basioccipital bone and no surgical complications occurred in the removal of the foreign bodies. Our technique used a variation of the approach for the modified (paramedian) ventral approach to the atlanto-axial joint as described by Shores and Tepper (9). This allows a direct approach but without the tedious dissection around vital structures when a straight midline approach is used. In addition, with this modification there is no need to transect the right stenothyroideus muscle nor to ligate the right cranial thyroid artery.

Certainly, a foreign body located in this area of the central nervous system is highly unusual. It is suspected that the two-halves of the needle shaft migrated through a penetration in the oral cavity and into the base of the caudal portion of the calvarium; however, no evidence of an oropharyngeal wound was found. The clinical signs and the radiographs identified the inciting cause; however, the exact location of the foreign bodies required advanced imaging (CT) and a 3D printing of the cervicomedullary junction to better formulate a surgical approach. The authors conclude that the modified approach (paramedian approach for ventral craniectomy) offers an improved method of exposing the basioccipital bone with less dissection and without performing any myotomies or ligation of arteries.

## Data availability statement

The original contributions presented in the study are included in the article/supplementary material, further inquiries can be directed to the corresponding author.

## Author contributions

OT-T performed all clinical aspects of this case including the examination, diagnostics, surgery, and aftercare. AS provided consultation, design of the surgical approach, and wrote the majority of the manuscript. Both authors contributed to manuscript revision, read, and approved the submitted version.

## Footnotes

^1^ANYCUBIC 3D Resin Printer, HongKong ANYCUBIC Technology Co, LTD., Hong Kong, China.

^2^Hall 5058-01 Surgairtome 2, Linvatec Corporation, Largo, Florida, United States.

^3^Gelfoam^®^, Pfizer Corporation, NY, NY, United States.
